# Response under low-energy electron irradiation of a thin film of a potential copper precursor for focused electron beam induced deposition (FEBID)

**DOI:** 10.3762/bjnano.9.8

**Published:** 2018-01-05

**Authors:** Leo Sala, Iwona B Szymańska, Céline Dablemont, Anne Lafosse, Lionel Amiaud

**Affiliations:** 1 Institut des Sciences Moléculaires d’Orsay (ISMO), CNRS, Univ Paris Sud, Université Paris-Saclay, F-91405 Orsay, France; 2 Department of Chemistry, Nicolaus Copernicus University in Toruń, Gagarina 7, 87-100 Toruń, Poland

**Keywords:** amines, copper(II), electron-stimulated desorption, FEBID precursors, HREELS, low-energy electrons, perfluorinated carboxylates

## Abstract

**Background:** Focused electron beam induced deposition (FEBID) allows for the deposition of free standing material within nanometre sizes. The improvement of the technique needs a combination of new precursors and optimized irradiation strategies to achieve a controlled fragmentation of the precursor for leaving deposited material of desired composition. Here a new class of copper precursors is studied following an approach that probes some surface processes involved in the fragmentation of precursors. We use complexes of copper(II) with amines and perfluorinated carboxylate ligands that are solid and stable under ambient conditions. They are directly deposited on the surface for studying the fragmentation with surface science tools.

**Results:** Infrared spectroscopy and high-resolution electron energy loss spectroscopy (HREELS) are combined to show that the precursor is able to spontaneously lose amine ligands under vacuum. This loss can be enhanced by mild heating. The combination of mass spectrometry and low-energy electron irradiation (0–15 eV) shows that full amine ligands can be released upon irradiation, and that fragmentation of the perfluorinated ligands is induced by electrons of energy as low as 1.5 eV. Finally, the cross section for this process is estimated from the temporal evolution in the experiments on electron-stimulated desorption (ESD).

**Conclusion:** The release of full ligands under high vacuum and by electron irradiation, and the cross section measured here for ligands fragmentation allow one to envisage the use of the two precursors for FEBID studies.

## Introduction

**Figure 1:**
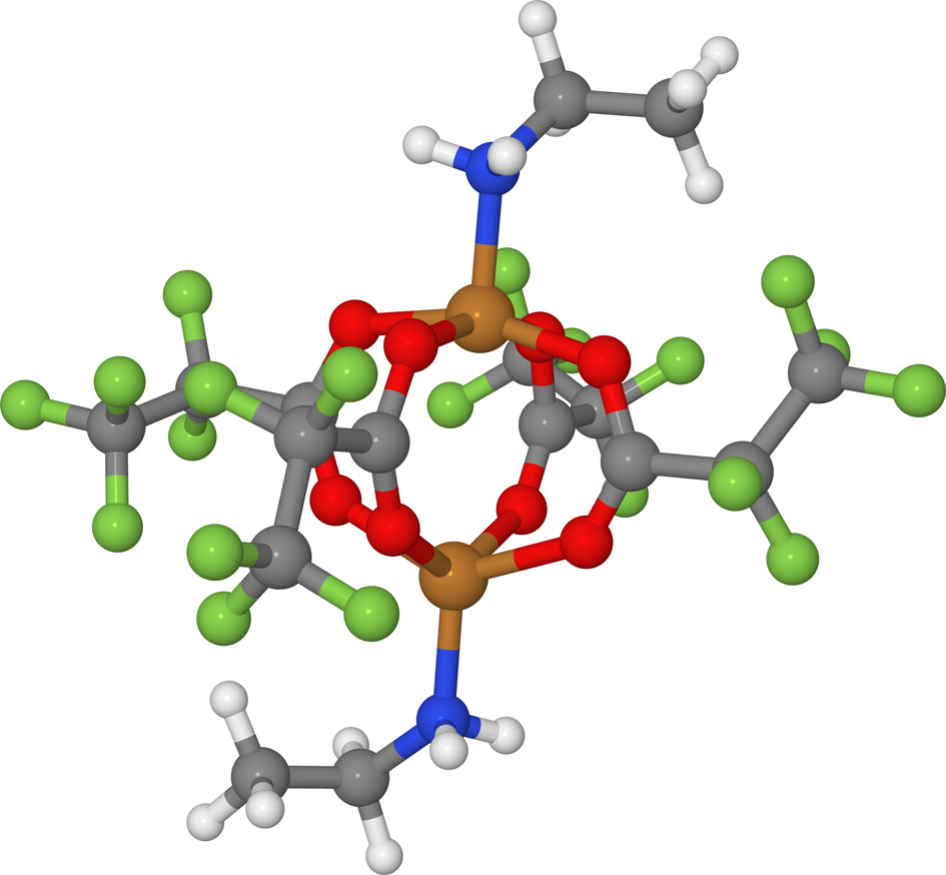
Schematic view of compound B, [Cu_2_(EtNH_2_)_2_(μ-O_2_CC_2_F_5_)_4_]; (H in white, C in grey, N in blue, O in red, F in green, Cu in brown).

The high electrical conductivity of copper makes it a material of choice for the development of electronic devices at the nanometre scale. In such applications, the conductivity of the deposit and its thermal behaviour strongly depend on the achieved purity, which still needs to be improved with standard techniques [1–2]. Pure deposits have been obtained with non-standard processes, for example with FEBID in aqueous solution [3] or with ion beam assisted deposition with plasma treatments [4]. For purity improvement of copper deposits, an alternative is the use of precursors with multiple copper ions. This class of metallic complex precursors has been proposed in situations where a good control of the composition is needed, for example in the case of alloy deposition [5]. A new group of copper precursors [Cu_2_(R′NH_2_)_2_(μ-O_2_CR)_4_] synthetized with two copper(II) cations has been designed by the co-authors at Nicolaus Copernicus University in Toruń, Poland, for chemical vapor deposition (CVD) [6–8]. Among these complexes, two different compounds, [Cu_2_(EtNH_2_)_2_(μ-O_2_CC_3_F_7_)_4_] and [Cu_2_(EtNH_2_)_2_(μ-O_2_CC_2_F_5_)_4_] (Figure 1) will be studied in the present paper and hereafter named as compound A and compound B, respectively. They differ only by the length of the four perfluorinated carboxylate ligands.

The use of lightly bound amine and carboxylate ligands gives complexes that are stable in gel form under ambient conditions but yield a copper purity from 60% to 80% in CVD. In these CVD studies, the sublimation of the full complexes was observed to be competitive with thermolysis of the ligands. The possibility to evaporate the compound allows one to envisage its use as a FEBID precursor. Moreover, the dissociation of the precursor to a pure heavy deposit and the release of volatile fragments is a crucial issue in FEBID. Considering the amine and carboxylate ligands, one can anticipate the ease of removal of the lightly bound amine ligands. In addition, the carboxylate ligands could favourably drive the precursor dissociation to the release of CO_2_ fragments, known to be a general product of electron impact on carboxylic acids [9–10]. These assumptions need to be confronted to experiments, not only for the specific use of the precursor in the present study, but also in order to contribute to the fundamental understanding of precursor dissociation in FEBID.

The evaporation of the precursor is a delicate aspect of FEBID experiments. The precursor should be not only in the gas phase, but also it needs to be deposited on the surface where the electron beam is focused. The main motivation of the present study is to get insights on the response of this precursor under FEBID conditions, i.e., under electron irradiation in vacuum. We separate here the deposition and the electron irradiation steps, in an approach similar to successful studies of different precursors deposited on surfaces and irradiated by 500 eV electrons [11–12]. To this end, one can avoid the challenging evaporation step and directly deposit the precursor on the surface before introduction into vacuum, like the work on Cis-platin particles by Warneke and co-workers [13]. We propose here a similar approach in the apparatus in Orsay devoted to the study of surface processes induced by low-energy electrons in the 0–20 eV range. This range is of particular interest as the low-energy electrons are known to play a major role in the chemical processes induced by energetic beams [11].

With a fixed precursor quantity, two particularities have to be noticed. Firstly, the temporal evolution of the recorded signals is mainly due to the changes in composition or quantity of the available precursor in the irradiation area. Secondly, irradiation is done in the beginning of the experiment in the electron-limited regime when the precursor quantity exposed to electron beam is not limited by adsorption and diffusion. This regime is known to give the best results in terms of spatial resolution [1] and therefore is of particular relevance for future use in FEBID.

In the first part of the present paper, a good stability of the complexes during transport from Poland to France is shown without excluding a slight loss of amine ligand. However, the loss of these amine ligands is enhanced by mild annealing in vacuum. In the second part, the fragmentation of the amine ligands under electron impact is discussed, as well as the fragmentation of the perfluorinated carboxylate ligands. The effective cross section for the fragmentation and release of these ligands under 1.5 eV electron impact is then finally estimated.

## Results and Discussion

### Stability of the EtNH_2_ ligands

**Figure 2:**
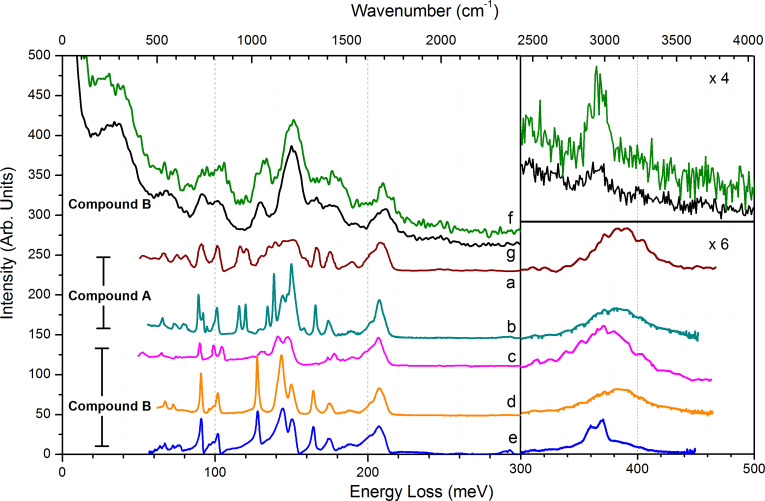
Vibrational signatures of compound A and B in different environments. (a, brown curve): ATR-IR spectrum of compound A as produced in Poland; (b, cyan curve): ATR-IR spectrum of compound A in France; (c, magenta curve): ATR-IR spectrum of compound B as produced in Poland; (d, orange curve) ATR-IR spectrum of compound B in France; (e, blue curve): ATR-IR of compound B on Si deposited by dip-coating; (f, green curve): HREELS spectrum of compound B deposited in gel form on gold, recorded at 30 K; (g, black curve): same as (f), after a mild annealing to 305 K.

Figure 2 shows the vibrational spectra of the two products recorded by different instruments: at ambient pressure by attenuated total reflection (ATR) infrared absorption spectroscopy (both in Poland and in France) and under vacuum by HREELS. The assignments of the main vibrational modes are listed in Table 1. The comparison between these signatures shows that the complexes, stable at atmospheric pressure in cold and dry environment, evolve when exposed to vacuum and when annealed under vacuum. The decomposition under heating has already been studied by variable temperature infrared spectroscopy (VT-IR) [8]. The effect of vacuum exposure is detailed hereafter.

**Table 1:** HREELS vibrational signature attributions.

vibrational modes^a^	energy loss		
	this work		reference
	meV	cm^−1^	meV

collective modes	37	298.5	
ν(Cu–N)	68	548.5	57^b^, 63^c^
ν(Cu–O_4_)	73	589	74^b^_,_ 61^d^
δ(CF_3_)	91	734	90^b^, 86^c^
δ(COO)	100	807	86-100^c^, 87^d^
ν(CC), ν(C–CN)	100–120	807–968	106^b^; 100–110^c^, 116–125^e^
ρ(CH_3_), ν(CN)	125–130	1008–1049	124^b^, 130^c^, 126^e^
ν(CF), ν(C–CF*_x_*)	115–165	928–1330	128–165^b^, 149–153^c^, 111–161^f^
ν_s_(COO)	177	1428	174^b^, 180^c^, 176^d^
δ(CH_2_), δ(CH_3_)	180	1450	180^g^
δ(NH_2_)	191	1541	189^b^
ν_a_(COO)	210	1694	206^b^, 219^c^ ν(C=O), 192^d^
ν(CH)	360–380	2900–3064	367^b^
ν(NH)	377–420	3040–3400	377–381^b^

^a^ν,ν_s_,ν_a_: stretching, symmetric, antisymmetric, δ: angular deformation, ρ: rocking; ^b^VT-IR, [Cu_2_(*t*-BuNH_2_)_2_(μ-O_2_CC_2_F_5_)_4_], [Cu_2_(EtNH_2_)_2_(μ-O_2_CC_2_F_5_)_4_] [7–8]; ^c^HREELS (5eV, specular), solid CF_3_COOH condensed at 30 K [10]; ^d^HREELS, C_6_H_5_COO^−^ chemisorbed on Cu (<1 ML, 8 eV, specular) [14]; ^e^Raman spectroscopy on a cysteamine self-assembled monolayer on silver [15]; ^f^IR of heptafluorobutyric acid [16], alkanethiol by HREELs [17].

### Amine ligand stability under ambient conditions

The IR spectra of the pristine compounds A and B are recorded in Poland (Figure 2, respectively, spectrum a and c). Compound A and B exhibit very similar vibrational signatures. Ethylamine apical ligands are visible by the signatures of the ν(CH) stretching band 360–380 meV, and the ν(NH) stretching band, positioned in the 380–400 meV range due to the coordination by the nitrogen group [18]. Additional small signatures of NH_2_ scissoring at 190 meV and ν(CC) in C–CN chains are seen at 100 meV and as a shoulder at 120 meV [8,15]. The four perfluorocarboxylates ligands are visible with the ν(COO) stretching at 206 meV, and the strong bands of mixed CC and CF_3_ stretching modes from 111 to 165 meV. This spectral range contains the main differences between the two compounds. The stability of the compound during the transfer from Poland to France under ambient conditions was verified by recording their IR spectra again (Figure 2, spectrum b and d).

Spectrum e in Figure 2 was taken with ATR-IR spectrometer in Orsay, France, probing compound B dip-coated on a Si wafer (thickness ca. 5 nm). Comparison of spectrum a with spectrum b, and of spectrum c with spectrum d shows that, for both compounds, most of the above mentioned signatures are still visible. Amine ligands are visible through the NH_2_ scissoring at 190 meV and mixed CH and NH stretching bands (350–400 meV). Only these last bands, especially the stretching NH contribution in the high-energy range, are visibly reduced on the dip-coated Si wafer, with nevertheless a significant remaining quantity.

### HREELS analysis of vacuum exposure and annealing effect

The sample used to record spectrum e in Figure 2 was introduced under vacuum for electron-stimulated desorption (ESD) experiments discussed in the next section, but HREELS analysis of these samples was not possible probably due to the combination of native oxide layer and the compound layers on it. Indeed, probing a poorly conductive sample in HREELS is always challenging. However, it was possible to probe a part of the silicon surface in a corner without deposited material. The spectrum showed a small signature of THF, which was the solvent used in the dip-coating process (not shown). Therefore, we changed for a conductive sample and avoided the THF signature in HREELS by direct deposit of the undiluted compound.

The vacuum vibrational analysis of the complex was done with a thin layer of compound B deposited on a bare gold surface (Figure 2, HREEL spectrum f). The spectrum was recorded at 30 K in order to improve the spectral resolution, even though it still remained much broader than with IR (ca. 7.6 meV in HREELS, 0.2 meV with IR). The spectrum shows broad and overlapping signatures that well enclose the signatures observed with IR in air, mentioning that relative intensities can vary from IR to HREELS. There are nevertheless important points to notice. The broad peak over the range of 350–400 meV is low compared to the usual signature of hydrocarbon layers in HREELS. This shows that an important fraction of the amine ligands is lost, in accordance with the absence of ν(NH_2_) stretching, expected as a weak and broad signature at 380–400 meV. Even if NH stretching modes are known to be difficult to observe in HREELS [19], the loss of amine ligands can be concluded from other spectral signatures. The δ(NH) angular deformation, the other expected contribution that is usually more intense, cannot be distinguished here at 192 meV, neither can the ν(C–CN) stretching at 115–120 meV. The signature of ν(CC) stretching at 130 meV cannot be unambiguously attributed to amine ligands as the fluorocarbon ligands have signatures in the same range. Only the shoulder at 180 meV, attributed to δ(CH_2_) and δ(CH_3_), are specific here to the amine ligands. In summary, all signatures of the ethylamine ligands are particularly small in spectrum f. The ν(CH) stretching and δ(CH) deformation attest for the remaining presence of amine ligands.

Further investigation of the compounds ability to lose its amine ligands was done by heating. Spectrum g in Figure 2 was recorded under the same conditions as spectrum f, but after a mild annealing above room temperature (*T* = 305 K), desorption was induced, visible by a small pressure rise in the chamber during the process. Back to low temperature, spectrum g is similar to spectrum f except that CH signatures are reduced (these signatures are mainly ν(CH) stretching, the shoulder of δ(CH_2_) and δ(CH_3_) at 180 meV, and the ν(CC) stretching shared with CF chains). Exposure to vacuum with mild annealing to 305 K leads to desorption of the amine ligands. This partial ligand loss from the complex is not a surprise as it was observed for a similar compound ([Cu(*t*-BuNH_2_)_2_(μ-O_2_CC_2_F_5_)_4_]) at atmospheric pressure, in the range 303 to 373 K, while the complex sublimation is observed between 413 and 473 K and the ﬂuorinated species decompose between 373 and 473 K [8]. In the VT-IR experiments, the vapour of the ([Cu(*t*-BuNH_2_)_2_(μ-O_2_CC_2_F_5_)_4_] compound was observed from 463 to 483 K. The characteristic bands for the decomposition products (CO_2_, anhydride, esters) were also detected.

### Fragmentation by low-energy electron irradiation

#### Electron-stimulated desorption

Further analysis of the precursor decomposition was done by ESD experiments on dip-coated samples (ca. 10–20 nm) of compound A_,_ introduced under vacuum at room temperature. Neutral fragments released during irradiation are detected with a quadrupole mass spectrometer (QMS). Neutral fragments can be released during irradiation by different processes. The most efficient process at high energy is the dissociative ionization (DI), above the ionization threshold. It produces a neutral fragment and a cation, which can be eventually neutralized. At lower energy, and above the electronic excitation threshold, the most efficient process is neutral dissociation (ND), which produces two neutral fragments. It is in competition with dipolar dissociation (DD), which produces two ions of opposite charges that can be further neutralized. In the low-energy range, below the two previously mentioned thresholds, the only efficient process is dissociative electron attachment (DEA), which produces neutral species and an anion that can be neutralized. ESD efficiency is higher at high energy because DI is the most efficient process. However, in FEBID experiments, the distribution of secondary electrons strongly peaks in the range of 1–5 eV, considerably enhancing the probability of DEA.

Moreover, the ESD experiments presented here were done at 300 K and are more susceptible to detect light species. At this temperature, heavier fragments can remain trapped by physisorption on the surface, but lighter neutral species would desorb, except those with strong dipolar momentum. In addition, the QMS detection yield is higher for light species. To put it in a nutshell, we cannot detect all fragmentation channels by ESD, and we suffer from a detection bias towards light species. Nevertheless, a fragment detected by ESD indicates a fragmentation channel likely to occur during focused electron beam irradiation, especially if its desorption is high in the range of 0–5 eV .

**Figure 3:**
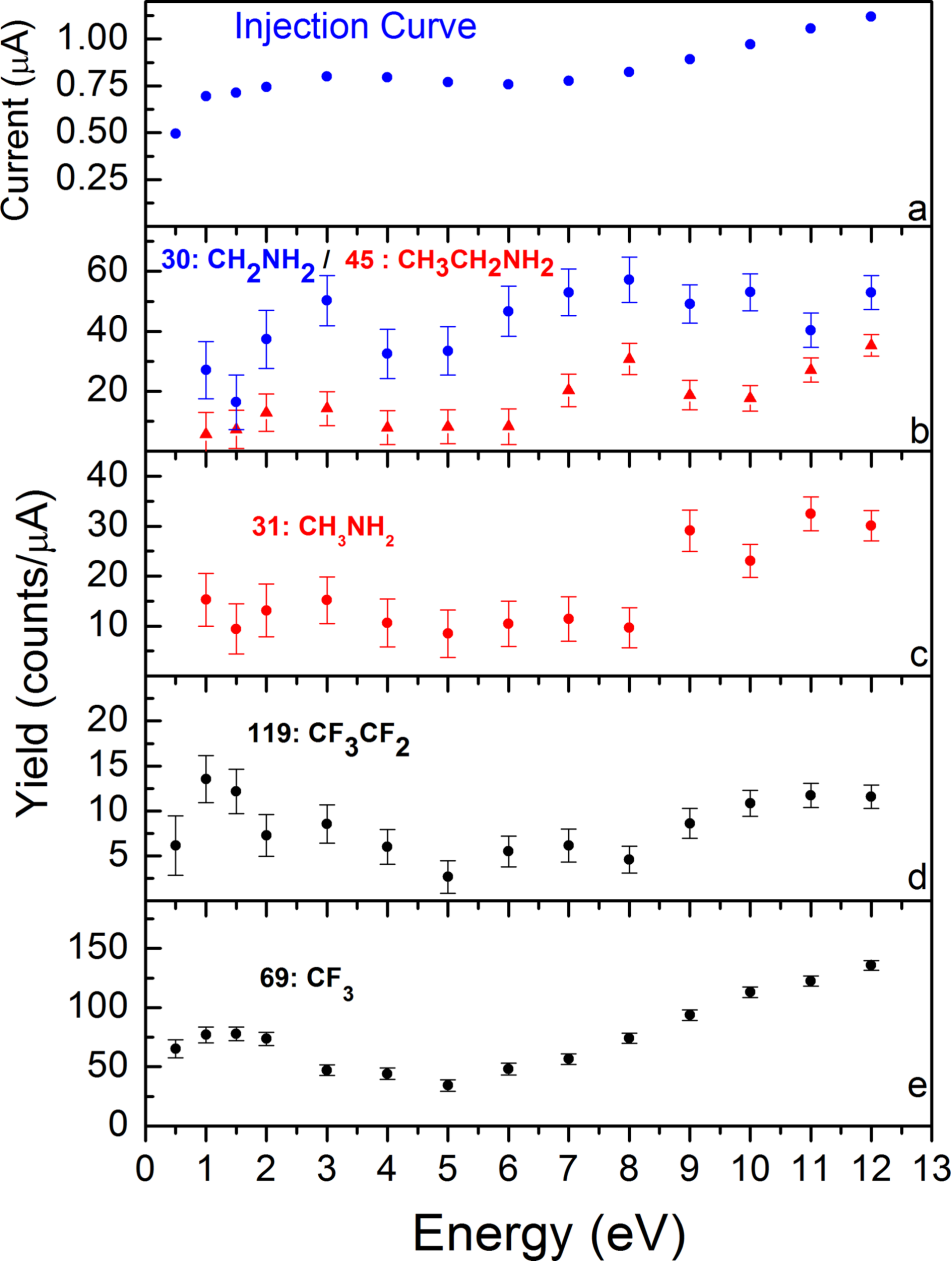
ESD experiments on compound A deposited on a Si wafer. (a) Mean current measured during irradiation; (b–e) recorded signal for neutral species detection, respectively, mass 30 (blue) and 45 (red), mass 31 (red), mass 119 (black), mass 69 (black). Most probable attributions are given in the corresponding colour.

The ESD spectra of selected fragments are presented in Figure 3. Figure 3a is used for the energy calibration (see Experimental section). Figure 3b shows the detection of neutral species of mass 45 and 30, respectively attributed to the whole EtNH_2_ molecule and its fragmentation in the QMS ionization head [20]. Their detection proves that the self-decomposition of the complex under vacuum at room temperature was incomplete and can be further induced by low-energy electron impact. Figure 3b shows that the stimulated desorption yield for neutral EtNH_2_ increases from 2 eV up to 12 eV. This global increase is the consequence of the successive dissociative channels, DEA, ND and DI, opening stepwise with the increase of the incident energy. In particular, the onset at 2 eV, which is low compared to the electronic excitation energy and ionisation energy of primary amines (respectively, 5.9 eV and 8.8 eV [21–22]) points out possible resonances of dissociative electron attachment processes leading to fragmentation of neutral ethylamine. The nature of the resonance is difficult to determine from the data. To our knowledge, no measurement of amine metal cation complex dissociation is described in literature for comparison, and the resonances for dissociative electron attachment in amine compounds, known from gas-phase studies [23–26], are expected to be shifted in energy and to have enlarged widths in condensed phase [27]. One cannot go further in the attribution based only on our measurements and the results of gas-phase studies. Nevertheless, the release of the ethylamine compound is favourable to the use of the compound as a precursor for FEBID. It corresponds to the loss of an entire ligand, which is certainly the most favourable situation to achieve improved purity of the final deposit. On the contrary, the ligand fragmentation can be viewed as a potential source of carbonaceous contamination of the future deposited product. The amine ligand fragmentation under electron irradiation is probed through the species with mass 31 (Figure 3c). This mass is not related to ethylamine fragmentation in QMS head [20]. Mass 31 can be taken as a good tracer of the amine ligand dissociation induced on the surface by electron impact. Its detection, attributed to CH_3_NH_2_ radicals, shows that the electron impact fragmentation of ethylamine on the surface has an onset at 8–9 eV, which is in agreement with the typical onset (8 eV) observed for carbon chain fragmentation in hydrocarbon films [28–29]. The fragmentation yield increases then with the electron energy, as the non-resonant processes, dissociative ionisation and electronic excitation followed by dipolar or neutral dissociation, become more efficient with increasing energy.

#### Fragmentation of perfluorocarboxylate ligands

The perfluorobutyrate ligand desorption induced by electron impact is observed by the detection of mass 119 and mass 69, which are attributed to CF_3_CF_2_ and CF_3_ radicals, respectively (Figure 3, curves d and e). The detection of the entire C_3_F_7_COO ligand and C_3_F_7_ fragments was not possible due to the low sensitivity of the mass spectrometer to high-mass fragments. Fluorocarbon desorption starts at the lowest energies, with a first maximum at 1.5 eV, then shows a minimum at 5 eV before rising again from 6 eV until the end of the scan at 13 eV.

Fluorocarbons are known to be cleaved through DEA [30–32], with a main feature of F^−^ anion production located at 4 eV in gas phase. The dependence on the energy is more structured in condensed phase, with three maxima at 3, 5 and 9 eV for the F^−^ desorption from condensed C_2_F_6_. Here, the 1.5 eV peak dominates the ESD curves for both CF_3_ and CF_3_CF_2_ desorption in the low-energy range. Considering the energy distribution of the secondary electron production in high-energy irradiation beams, which strongly peaks at very low energy [33–34], an efficient dissociation process in this range would have strong consequences on chemistry induced by irradiation. The nature of the dissociation process is most probably due to dissociative electron attachment, which is the only efficient process at low energy. Attribution based on the position of the desorption maximum is tentative. We can nevertheless note the accordance of these results with the well-known resonance of dissociative electron attachment on small carboxylic acids in the gas phase [9,35], observed also in the condensed phase [10,29]. It has been shown that carboxylic acid has a resonance for electron attachment centred at 1.5 eV, leading to atomic rearrangement and breaking of the R–COOH bond, visible by the formation of CO_2_ and H_2_O in the condensed phase. Here, the carboxylates are coordinated to Cu^2+^ cations. It would be interesting to investigate if the low lying shape resonance of π* character near 1 eV, involved in the dissociative electron attachment process on carboxylic acids, is preserved in a coordination complex and could lead to the cleavage of the Cu–OOCR coordination bond. Unfortunately, CO_2_ formation, which could have given us more information on the dissociation processes, could not be observed. The signal for the detection of CO_2_, or the secondary fragment CO, did not rise sufficiently from the residual background noise.

#### Estimation of the effective cross section for carboxylate ligand loss

**Figure 4:**
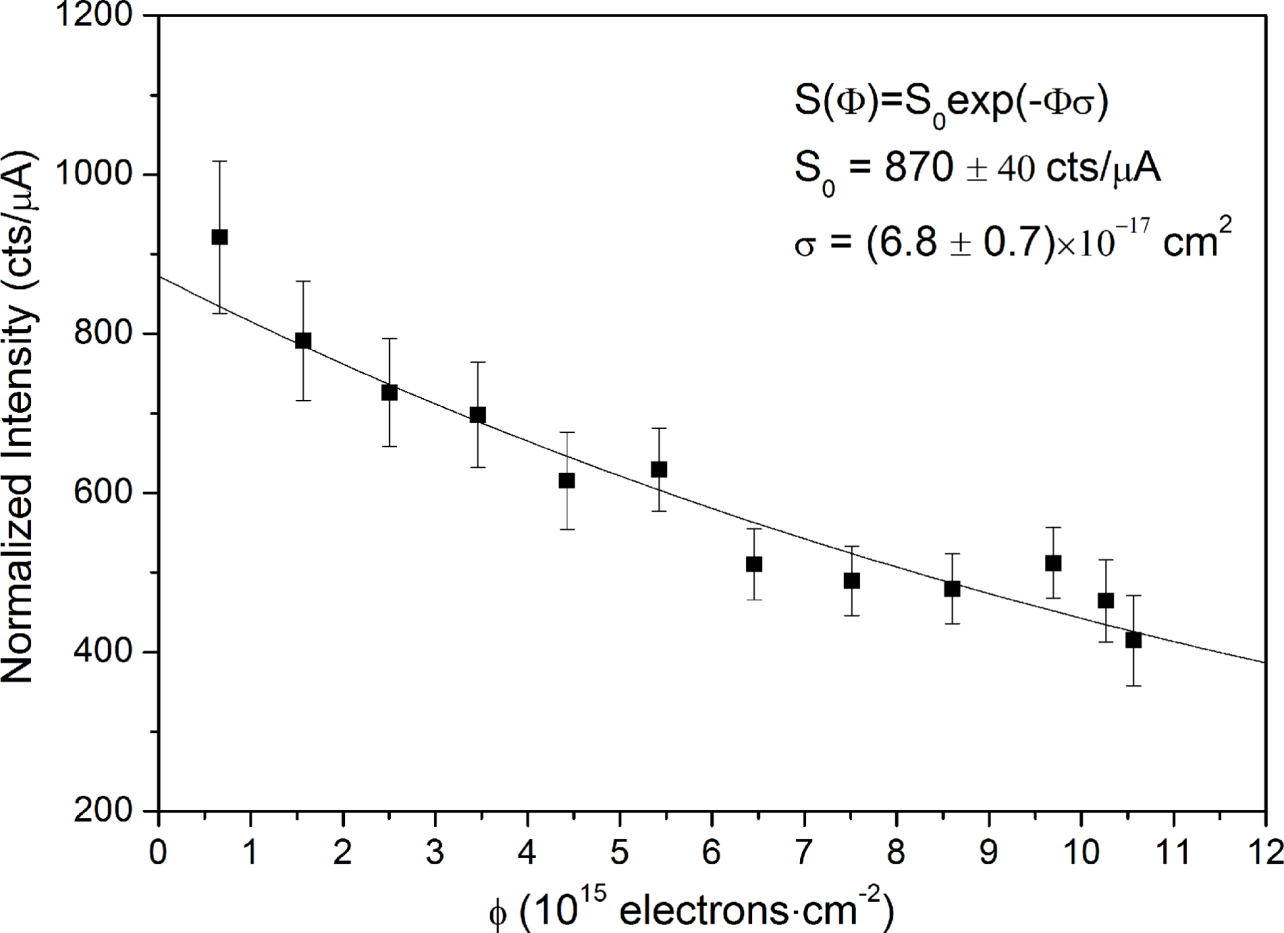
Evolution of CF_3_ neutral fragment detection with the cumulated dose (fluence) under irradiation at 1.5 eV. Squares: measurement, line: adjusted model.

The analysis of the desorption signal for a long irradiation time allows to estimate the efficient cross section for the chemical transformation induced all along the irradiation. A dip-coated sample on silicon, with an estimated thickness of 5 nm, is exposed to a 1.5 eV electron beam for 210 min. Regular off-beam measurements are taken to remove the background contribution from the desorption signal. The evolution with the cumulated dose of the CF_3_ signal, normalized to transmitted current, is plotted in Figure 4. The signal is decreasing with time as the available quantities on the surface are decreasing. Assuming a direct proportionality between the signal and the remaining quantity on the surface, this measure can be used to estimate the effective cross section for the fragmentation and desorption of the carboxylate ligand by electron impact at 1.5 eV. We can assume that the mean free path of the electrons is long enough to interact with the whole 5 nm of available product [34]. All available complex material is then considered exposed to the irradiation beam from the beginning of the experiment. As already used in a previous publication [36], the decrease in the signal follows an exponentially decreasing law: 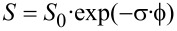 where *S*_0_ is the initial signal intensity, σ is the cross section (in cm^2^) and 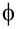 is the beam fluence (in cm^−2^), i.e., the integrated number of incident electrons over time. The desorption signal must decrease and drop to zero when the compound is fully consumed. Here, the experiment is stopped before the end, when more than 50% of the compound has been transformed and the layer starts to significantly change compared to the beginning of the measurement. A part of the complexes have lost their ligands and the cross section for the loss or the fragmentation of a second or third ligand is not expected to be the same. A fitting procedure using the first phase of the evolution curve allows to deduce the value of σ = 7 × 10^−17^ cm^2^. Comparing to data available in the literature, this value is larger than the cross section of electron attachment on acetic acid leading to its dissociation to CH_3_COO^−^, which is 6 × 10^−19^ cm^2^ in the gas phase [37]. This result is not surprising since, for the dissociation route RCOO^−^ + H solely, it has been observed that fluorinated molecules have a cross section of more than ten times greater than that of equivalent hydrogenated compounds [38]. The condensed compound here has also a longer carbon chain, fully fluorinated, and can have additional fragmentation routes not included in the gas-phase study. The cross section deduced here is 100 times smaller than the total electron scattering on formic acid, around 4 × 10^−15^ cm^2^ [9,39].

Considering the cross section obtained here, the fully coordinated complex, which contains four perfluorinated carboxylate ligands, has a cross section four times larger for the fragmentation of one ligand seen by CF_3_ desorption. It can be estimated to σ′ = 2.7 × 10^−16^ cm^2^. This is the order of magnitude of the cross section for NO ligand loss from [Co(CO)_3_NO] by dissociative electron attachment at 2 eV seen in the gas phase, (0.5–4.0) × 10^−16^ cm^2^ [40]. The loss of PF_3_ from [Pt(PF_3_)_4_] due to electron attachment at 0.5 eV, measured as 2 × 10^−16^ cm^2^ in the gas phase, is also within the same range [41].

## Conclusion

The present study is our first attempt to use surface science tools, usually dedicated to the study of low-energy electron induced processes, to gain insights on dissociation processes induced on potential precursors for FEBID. The HREEL spectroscopy allowed us to show that the precursor loses spontaneously the amine ligands when it is exposed to vacuum at room temperature. There is additional information that could be obtained from detailed HREELS studies. In particular, the evolution of the vibrational signatures with the incident energy of the probing electron beam can be used to find resonance energies for electron attachment [42]. Such energies can be probed to better understand the key role of DEA process in precursor decomposition under electron beams. For this purpose, the homogeneity of precursor deposition must be improved on a good conductive substrate to avoid partial charging of the system that complicated the HREELS investigation in the present study. ESD measurements have shown that the loss of the amine ligands was not completely achieved under vacuum at room temperature and can then be reinforced by electron bombardment in the low-energy range. Irradiation strategies under 5 eV could be considered in the aim of preserving ligand integrity in order to reduce carbon impurities in the copper deposit. This is for the moment beyond the possibilities of scanning electron beams because achieving both low landing energy and high current density remains a challenge [43–44].

Perfluorinated carboxylate ligands were observed to dissociate under electron irradiation. The neutral CF_3_ fragments were detected, and this detection shows that an efficient dissociation process occurs at 1–2 eV. At such low energy, this process is supposed to be of importance under FEBID conditions, considering that the energy distribution for secondary electrons emitted in the direct vicinity of the irradiation spot strongly peaks in the range of 1–5 eV, and can take part in the whole dissociation process. The effective cross section for CF_3_ release from the complex compound has been measured from evolution of the signal directly linked to the rarefaction of the precursor in the irradiation area.

## Experimental

The precursor in gel form can be directly deposited on a surface or can be diluted in solvent for a dip-coating deposition, which both allow the study by surface science tools in vacuum environment.

**Preparation of copper complex compounds:** [Cu_2_(EtNH_2_)_2_(μ-O_2_CR)_4_], R = C_2_F_5_ and C_3_F_7_, and of dip-coated samples were performed at Nicolaus Copernicus University, Toruń, Poland. Copper(II) carboxylate [Cu(O_2_CR)_2_] (1·10^−3^ mol for R = C_2_F_5_, C_3_F_7_) was dissolved in 20 cm^3^ acetonitrile, and ethyl isocyanate in 5 cm^3^ of acetonitrile (1·10^−3^ mol) was dropped. The obtained reaction mixture was stirred for 3 h, in air, at room temperature. Next, the solution was filtered, and the final product was isolated by solvent evaporation at reduced pressure, in argon. The obtained compounds were blue gels, stable in air, which should be moisture-protected during the storage. They were kept in cold and dry environment before use. The basic physicochemical data for [Cu_2_(EtNH_2_)_2_(O_2_CC_2_F_5_)_4_] are listed in [45]. For [Cu_2_(EtNH_2_)_2_(O_2_CC_3_F_7_)_4_], elemental analysis, EIMS and IR are listed below: Anal calcd. for C_20_H_14_Cu_2_F_28_N_2_O_8_: Cu, 11.8, C, 22.5; H, 1.32; found: Cu,12.2; C, 22.8; H, 2.11; EIMS (*T* = 417 K) *m*/*z* (% relative intensity): [C_2_H_7_N]^+^ 45 (7), [Cu_2_(EtNH_2_)(O_2_CC_3_F_7_)]^+^ 384 (2), [Cu_2_(O_2_CC_3_F_7_)]^+^ 339 (100); [Cu_2_(O_2_CC_3_F_7_)_2_]^+^ 552 (28), [Cu_2_(EtNH_2_)_2_(O_2_CC_3_F_7_)_3_]^+^ 855 (3); IR (KBr): 3063, 3014, 2924, 2844, 2744, 2608, 2504, 2098, 1679, 1528, 1479, 1405, 1338, 1218, 1164, 1119, 1083, 968, 934, 817, 743, 719, 650, 589, 529, 452, 421; PE 422, 394, 331 cm^−1^.

**Dip-coating:** Dip-coated samples were prepared in Toruń, Poland. Precursors were dissolved in tetrahydrofuran, forming an almost saturated solution, and deposited on Si(111). The dip-coating parameters were the following: the withdraw rate was 80 mm/min, the immersion rate was 80 mm/min, the immersion time was 20 s, and the coating count was 10. ATR-IR spectra were recorded with a PerkinElmer spectrum two instrument, equipped with a single reflection diamond crystal.

**Experimental setup in Orsay, France:** The experiment setup at ISMO in Orsay, France is detailed in previous publications [29,36,46]. Samples are mounted on the cold finger of the cryostat and their temperature can be controlled from 30 K to 750 K. The temperature is measured with a Pt103 sensor and a Lakeshore 335 controller with an accuracy of 1 K.

**HREELS:** HREELS measurements were carried out on a gold on glass sample, from Arrandee GMBH. The HREELS (model IB500 - Omicron with a control electronic by LKS technology) is housed in a custom-built apparatus with base pressure of 1.2·10^−10^ mbar. HREEL spectra are recorded at 30 K, with an incident energy *E*_0_ = 13 eV (FWHM = 7.6 meV). Typical current on the sample during HREELS record is 1 nA·cm^−2^ for 10 min, focussed on a 4 mm^2^ spot. The cumulated exposure by HREELS in this spot is 3·10^13^ electrons·cm^−2^_,_ which is 100 times smaller than during ESD experiments.

**Electron-stimulated desorption:** ESD experiments were carried out in a secondary chamber, accessible through a gate valve to HREELS, and with a load-lock entry. It houses an ELG2 electron gun (Kimball physics) and a quadrupole mass spectrometer (3F-PIC- Hidden Analytical). Neutral fragment desorption induced by low-energy electron impact is recorded following a procedure described in previous publications [29,36] improved here with the pulsing of the electron gun in place of the sample potential. The irradiation spot has 6 mm in diameter, with a current close to 1 μA. The electron energy is deduced from the potential difference between electron-gun filament and the sample. The rapid onset of the injection curve (Figure 3a), showing an abrupt start at zero, assures that we can set, in the resolution limit of our experiment, the equality between the real impact energy and this potential difference with an uncertainty of ±0.5 eV. During irradiations, different processes can occur and the resulting neutral fragments are detected with a QMS. The QMS head is optimized for low partial pressure detection of neutral species. Neutral fragments are ionized at the entrance of the QMS by electron induced collisions (at 70 eV), and eventually fragmented. The positive ions are then filtered according to the mass to charge (*m*/*z*) ratio and detected by a channeltron. The signal is attributed to neutral species, even if ions could be formed on the surface. Indeed, due to the potential geometry in the ionization head, the detection of primary ions coming from the surface is unlikely, and this can be confirmed by turning off the electron source of the QMS, nullifying the detection. Moreover, the charged species need to overcome an additional desorption barrier due to the mirror charge potential created on a conductive sample. The detection yields given here are normalized by the transmitted current on the sample but not corrected by the detection efficiency of the instrument.
